# A Comprehensive Overview of the Parathyroid Tumor From the Past Two Decades: Machine Learning-Based Bibliometric Analysis

**DOI:** 10.3389/fendo.2021.811555

**Published:** 2022-01-26

**Authors:** Zeyu Zhang, Fada Xia, Xinying Li

**Affiliations:** Department of Thyroid Surgery, Xiangya Hospital, Central South University, Changsha, China

**Keywords:** parathyroid tumor, bibliometrics, machine learning, *natural language processing*, parathyroidectomy

## Abstract

**Introduction:**

Parathyroid tumor, in particular carcinoma, is fairly rare among neoplasms of the endocrine system, unlike its benign counterpart. However, there is no bibliometric analysis in the field of parathyroid tumors comprehensively summarizing and discussing a large number of publications by a machine learning-based method.

**Materials and Methods:**

Parathyroid tumor-related publications in PubMed from January 2001 to December 2020 were searched using the MeSH term “parathyroid neoplasms”. Latent Dirichlet allocation was adopted to identify the research topics from the abstract of each publication using Python.

**Results:**

A total of 3,301 parathyroid tumor-associated publications were identified from the past 20 years, and included in further analyses. Research articles and case reports occupied the most proportion of publications, while the number of clinical studies and clinical trials decreased, especially in recent years. Technetium Tc 99m sestamibi was most studied among the diagnosis-related MeSH terms, while parathyroidectomy was among the treatment-related MeSH terms. The Latent Dirichlet allocation analyses showed that the top topics were ^99m^Tc-MIBI imaging, parathyroidectomy, gene expression in the cluster of diagnosis research, treatment research, and basic research. Notably, scarce connections were shown between the basic research cluster and the other two clusters, indicating the requirements of translational study turning basic biological knowledge into clinical practice.

**Conclusion:**

The annual scientific publications on parathyroid tumors have scarcely changed during the last two decades. ^99m^Tc-MIBI imaging, parathyroidectomy, and gene expression are the most concerned topics in parathyroid tumor research.

## Introduction

Parathyroid tumor (PT), in particular carcinoma, is pretty rare among neoplasms of the endocrine system, unlike its benign counterpart (1). Unlike thyroid cancer, which shows a predominance in women, parathyroid carcinoma has a consistent incidence in both sexes (2). While most PTs are sporadic, various genetic diseases may also cause PT, including hyperparathyroidism–jaw tumor syndrome and multiple endocrine neoplasias. The diagnosis of PT is often made by serum biomarkers and radiological examinations; however, it can be hard to preoperatively distinguish the malignancy from the adenoma. Surgery remains the only curative therapy of PT; however, recurrence is shown in 23%–50% of patients who previously received surgery (3).

Accumulative articles have reported significant progress and developments in the field of PT, and the research hotspots, as well as future research directions, can be reflected by these publications. Bibliometric analysis is often used to summarize the contributions of publications. To our knowledge, there is no bibliometric analysis in the field of PT, comprehensively summarizing and discussing a large number of publications.

Besides the regular bibliometric analysis methods, machine learning is also developed to analyze human language, such as natural language processing. Among these methods, latent Dirichlet allocation (LDA) is most frequently applied in the scientific publication analysis by identifying specific themes and dividing documents into these themes (4).

The objective of this study is to map the research landscape of PT through analyses of scientific publications in the past two decades. Furthermore, by applying a machine learning method, this study may also contribute to recognizing features of specific research topics in the field of PT.

## Materials and Methods

The MeSH term “parathyroid neoplasms” was used to identify PT-related publications in PubMed from January 2001 to December 2020. An R package “Bibliometrix” was used for extracting associated data (5), including the publication year, the publication type, MeSH terms, and abstract. To simplify the MeSH terms analysis, MeSH terms appearing less than 10 times were excluded. Additionally, ethical approval was waived because of the nature of the bibliometric analysis.

To recognize the research topics of each publication in detail, the abstract was analyzed by LDA using the Python platform. A feature glossary of terms was established by the coexistence frequency of vocabulary words in the publication series, and the two most probable research topics would be calculated for each publication, depending on the appearance frequency of these glossary words. Subsequently, the Louvain algorithm was applied for cluster analyses to clarify the associations between topics.

The R platform and Excel were applied for visualizations, while Gephi (https://gephi.org/) was adopted for the network construction (6). All the involved codes, including R platform and Python platform, were available on GitHub (https://github.com/yan-wen0614/Medicine-Bibliometric-Analysis).

## Results

**Figure 1:**
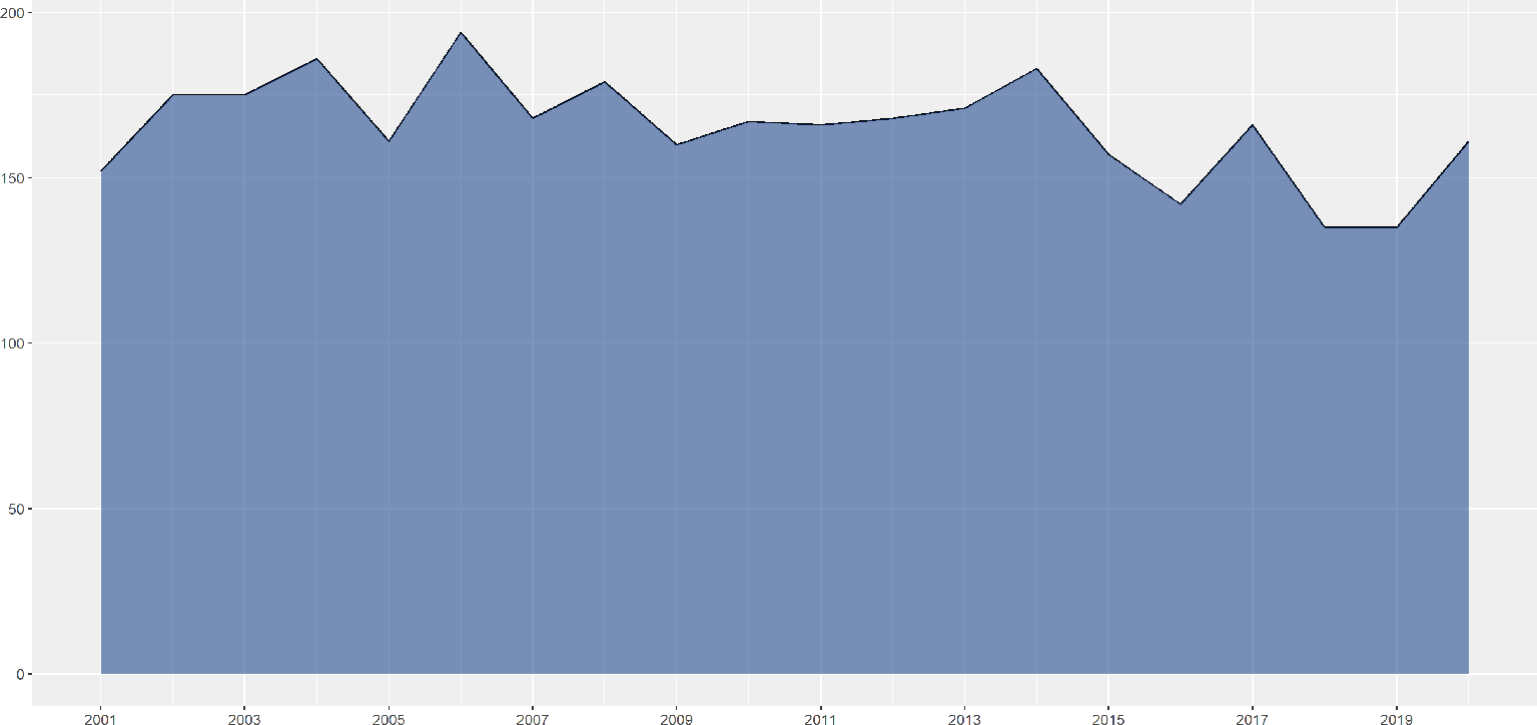
Scientific publications per year.

**Figure 2:**
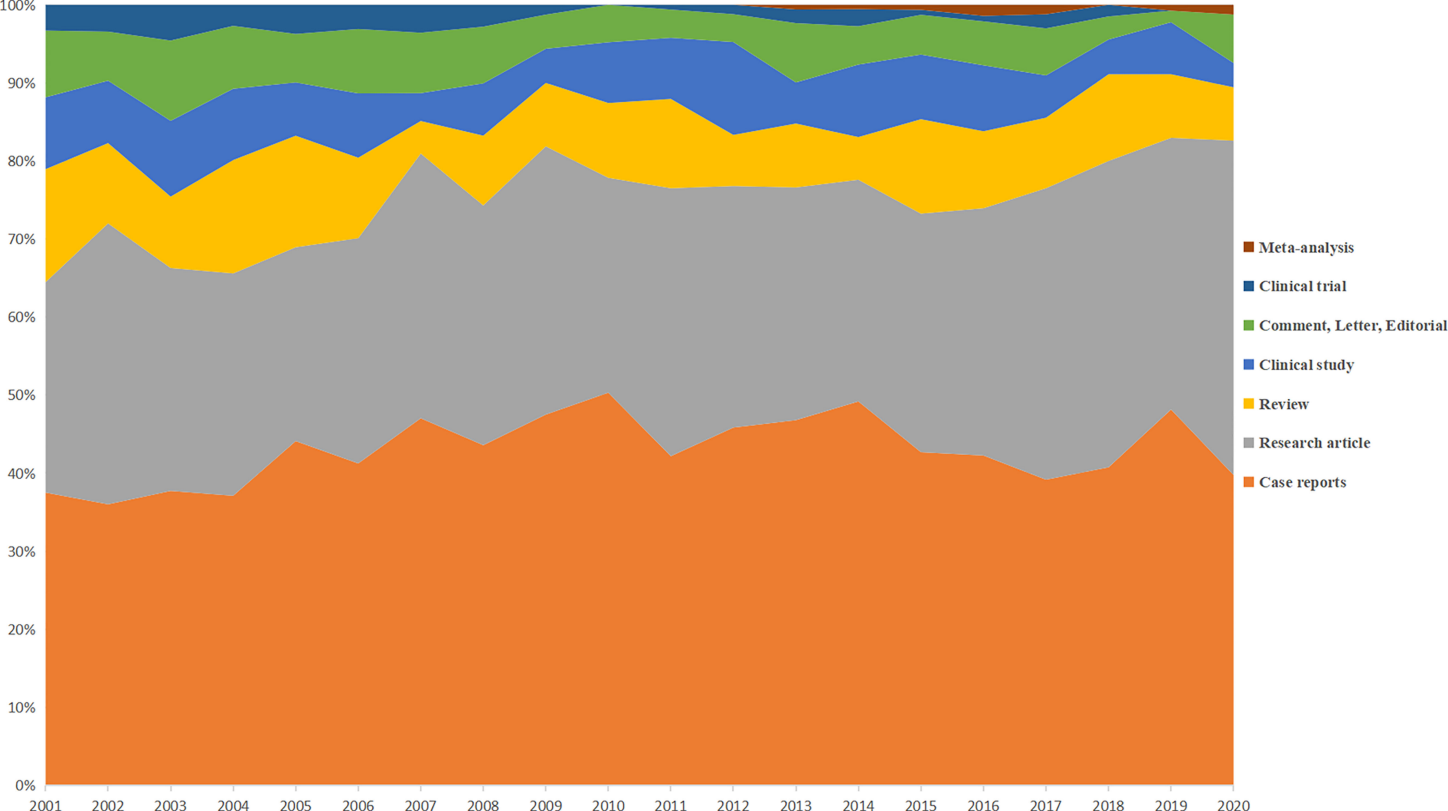
Distribution of publication types per year.

**Figure 3:**
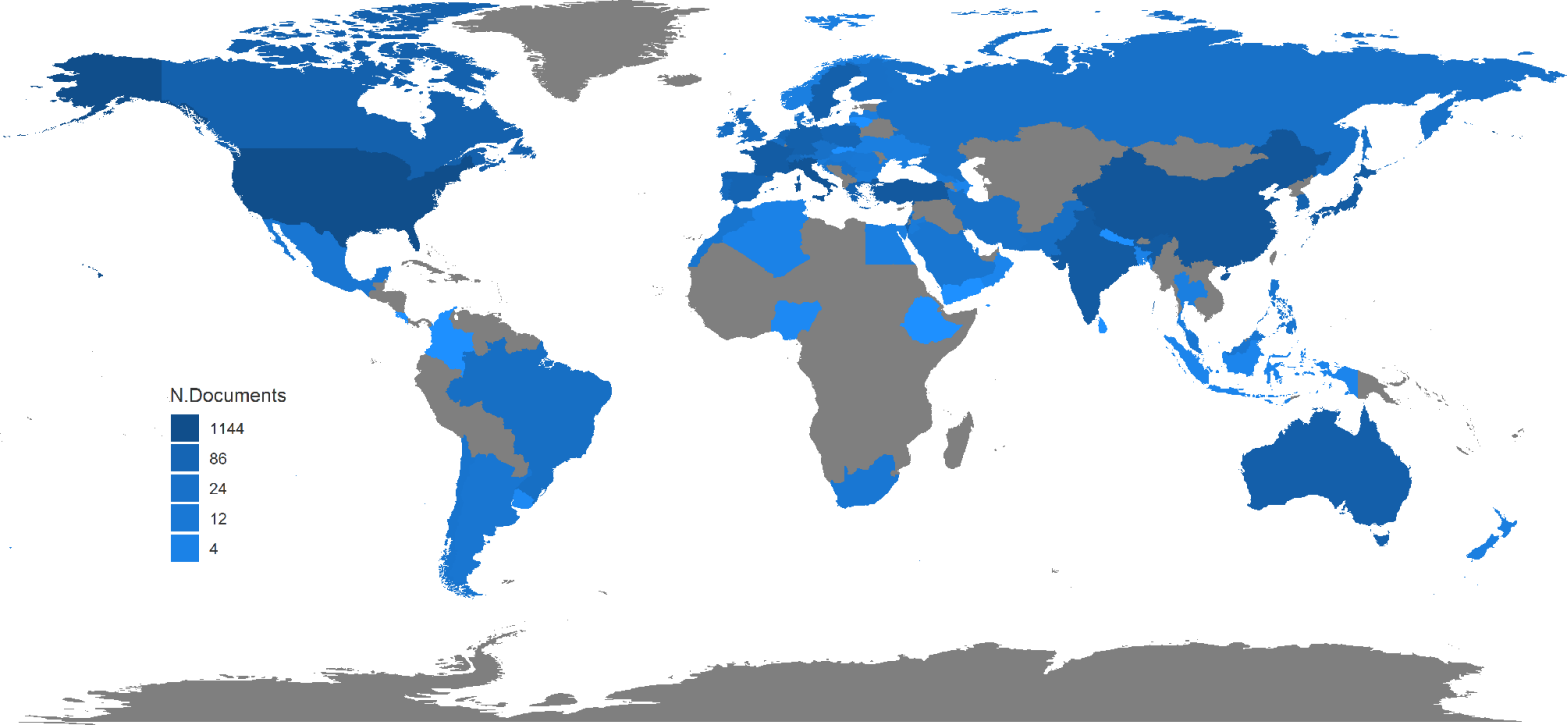
Country scientific production.

A total of 3301 PT-associated publications were identified from the past 20 years and included in further analyses. Even with the massive growth of overall scientific publications, **Figure 1** showed that the annual publication number remained scarcely changed during the past two decades with the highest as 194 in 2006 and the lowest as 135 in 2018. **Figure 2** showed the distribution of publication types. Research articles and case reports occupied the most proportion of publications, while the number of clinical studies and clinical trials decreased, especially in recent years. The PT-related meta-analysis was firstly shown in 2013, but without significant development, which may be caused by the decreasing number of clinical studies and trials. Furthermore, **Figure 3** shows the country of scientific production. Fifteen countries published more than 100 records, with the USA as the top one (1144 publications), followed by Italy (583 publications), China (452 publications), Turkey (317 publications), and India (262 publications). Additionally, **Table 1** listed the top 10 affiliations with the most contribution to PT-associated productions.

**Table 1:** Top 10 affiliations with the highest scientific production.

Name of affiliation	Number of articles
The University of Texas MD Anderson Cancer Center	94
Peking Union Medical College Hospital	94
University of Pisa	57
University of Connecticut School of Medicine	50
University of Wisconsin	50
Duke University Medical Center	49
Postgraduate Institute of Medical Education & Research (PGIMER)	48
Capital Medical University	46
Centre Hospitalier Universitaire De Nantes	44
University Of Milan	43

### MeSH Term Analyses

Further analyses were performed based on 438 MeSH terms found in obtained publications with a total of 31,588 times of occurrence.

**Figure 4:**
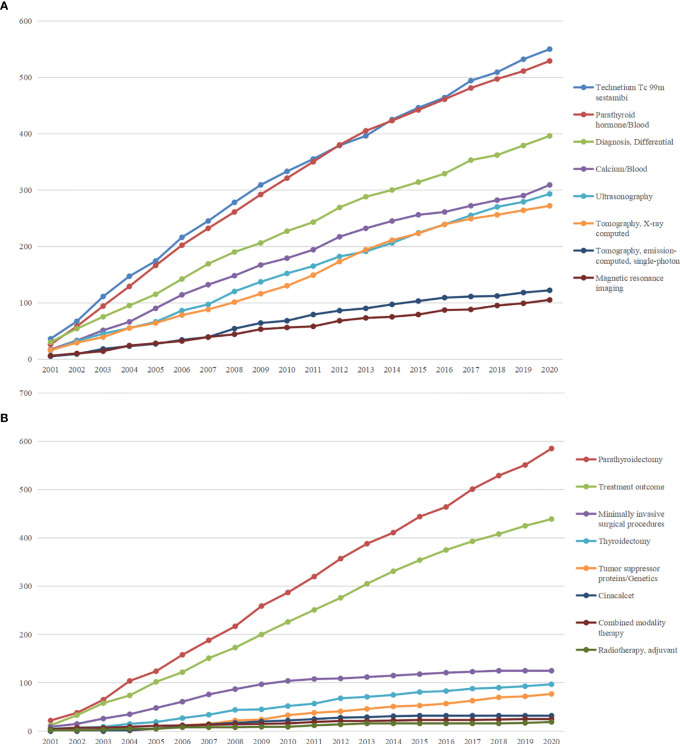
**(A)** Accumulative occurrences of top 8 MeSH terms concerning the diagnosis of parathyroid tumor. **(B)** Accumulative occurrences of top 8 MeSH terms concerning the treatment of parathyroid tumor.

**Table 2** showed some general issues of PT-associated studies, including study subject, age group, and study design. Notably, compared with human-based studies, the number of studies on animals and cells was very limited, suggesting potential vulnerabilities in comprehensive mechanism investigations. We subsequently investigated most focused diagnosis-related MeSH terms (**Figure 4A**) and treatment-related MeSH terms (**Figure 4B**). Among the diagnosis-related terms, technetium Tc 99m sestamibi was the most studied. Multiple imaging examinations showed significance in diagnosing PT, including ultrasonography, computed tomography (CT), single-photon emission computerized tomography (SPECT), and magnetic resonance imaging (MRI). Blood biomarkers, including parathyroid hormone (PTH) and calcium, were also frequently involved. In addition, differential diagnosis was another significant issue when diagnosing PT. In terms of treatments, parathyroidectomy was the most concerned therapy, while treatment outcome was the most concerning issue. Except for surgery, targeted therapy and radiotherapy were also developed to suppress PT, while combined modality therapy was under development. Moreover, cinacalcet was also highly concerned in PT-associated publications as an essential method of controlling blood calcium in advanced PT.

**Table 2:** Top three terms in general study issues of parathyroid tumor during the past 20 years.

Category	Name of affiliation	Number of occurrences
Study subject	Human	3,264
	Animal	91
	Cell	15
Age group	Middle aged	1,651
	Adult	1,292
	Aged	1,157
Study design	Retrospective studies	509
	Follow-up studies	208
	Prospective studies	138

### LDA Analyses

**Figure 5:**
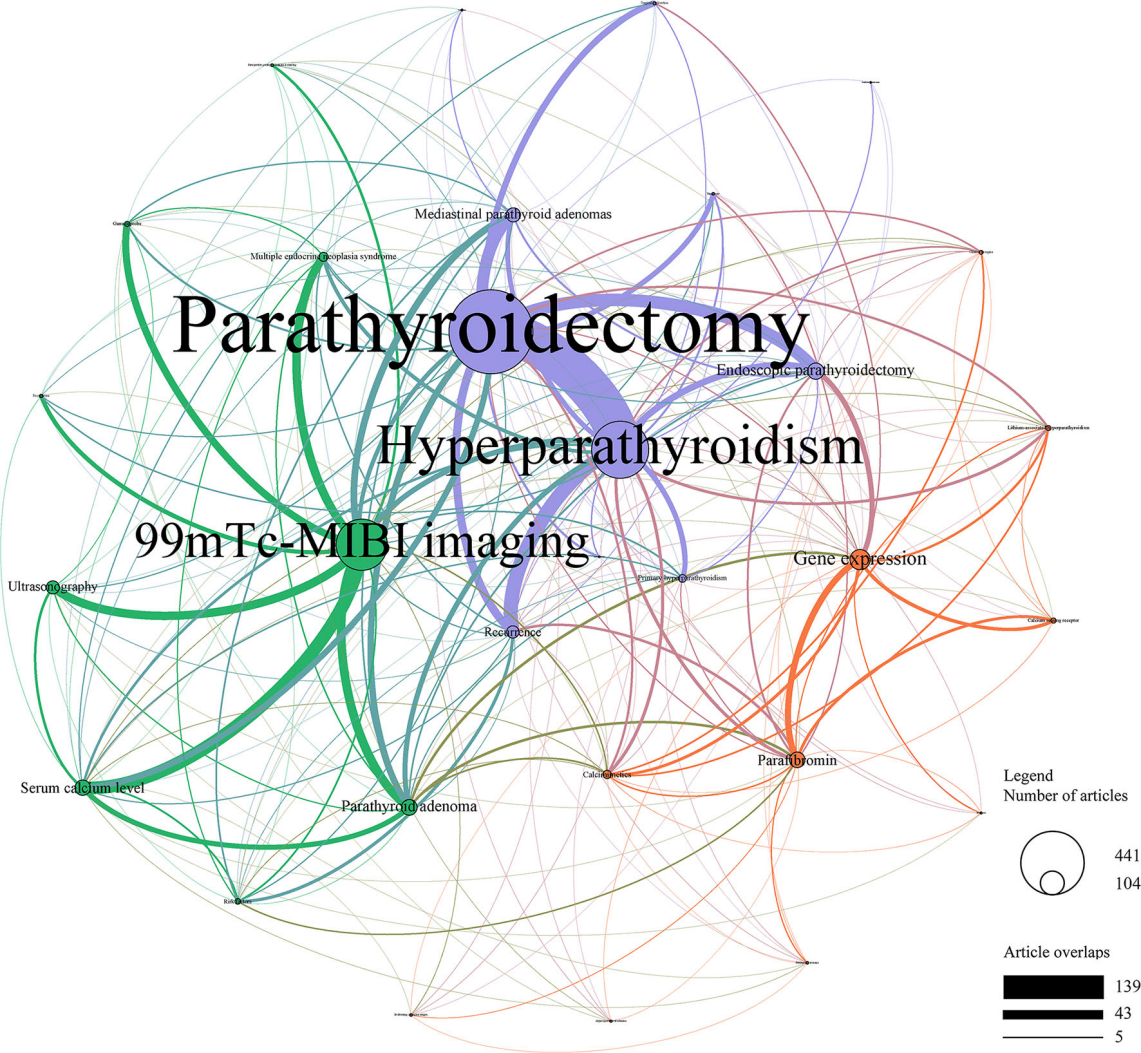
Topic cluster network by Latent Dirichlet Allocation. Green, Diagnosis research; Purple, Treatment research; Red, Basic research. The size of the circle represents the number of papers in each topic, and the thickness of the line represents the weight of the connection between each topic.

Further analyses were performed by a machine learning method (LDA) using abstracts from publications. While excluding 652 publications without an abstract, the 30 hottest research topics were extracted by LDA analyses using abstracts of the remaining 2,649 publications, and a topic network was built to illustrate these topics and associations between them (**Figure 5**). These topics were allocated into three clusters according to Louvain algorithm, including diagnosis research (in green), treatment research (in purple), and basic research (in red). The focalization of a topic and the weight of the connection between topics were also shown as the size of the circle and the thickness of the line, respectively.

In the cluster of diagnosis research, ^99m^Tc-MIBI imaging and parathyroid adenoma were the top two topics, emphasizing the value of differential diagnosis between parathyroid cancer and parathyroid adenoma. Serum calcium level and ultrasonography were also hot topics, which was consistent with the results of MeSH term analyses. Particularly, ^99m^Tc-MIBI imaging showed wide connections with other topics in the cluster of diagnosis research. In terms of treatment research, parathyroidectomy was the top topic, followed by hyperparathyroidism, endoscopic parathyroidectomy, mediastinal parathyroid adenomas, and recurrence. A strong connection was shown between parathyroidectomy and hyperparathyroidism. Furthermore, gene expression, parafibromin, and calcimimetics took the majority proportion of the basic research cluster. Notably, scarce connections were shown between the basic research cluster and the other two clusters, indicating the requirements of translational study turning basic biological knowledge into clinical practice.

## Discussion

For the very first time, this machine learning-based bibliometric study summarized 3301 publications of PT from the past two decades. Despite the rapid expansion of scientific publications, there was very limited change in the number of PT-related publications, suggesting more attention needs to be paid to the research field of PT. Meantime, the number of clinical studies or trials decreased in recent years, suggesting more attention is needed on the clinical management of PT. The most concerning research topics were ^99m^Tc-MIBI in the diagnosis section, parathyroidectomy in the treatment section, and gene expression in the basic research section. Meanwhile, more efforts should be paid to translation through preclinical researches to clinical practices. Overall, this study demonstrated patterns and trends of the past and prevailing topics in PT research, which may provide new in-depth directions for both researchers and practitioners.

The most frequently used imaging examinations for diagnosing PT are ultrasonography and ^99m^Tc-MIBI imaging. With a low economic cost and high practicability, ultrasonography plays an important role in preoperatively detecting PT. However, the poor capability of ultrasonography is shown in distinguishing the malignant from the benign (7). ^99m^Tc-MIBI imaging, including scintigraphy and SPECT, is also commonly used to identify PT based on the different retention levels. Although ^99m^Tc-MIBI imaging was recognized as a method without abilities of differential diagnosis for a very long time, recent research reported that the peak of retention index may contribute to the preoperative differential diagnosis of parathyroid malignancy (8). Moreover, choline PET is a novel diagnostic method for PT, which this study failed to highlight due to its novelty. It was reported to bear the potential as a significantly more sensitive method to replace traditional imaging (9). However, even with the development of novel diagnostic methods, different diagnosis still remains the priority issue in the area of PT diagnosis.

So far, surgery remains to be the best chance of cure for PT. The simple parathyroidectomy is considered suitable for most benign PT, while far from enough for parathyroid cancer. The gold surgical procedures contain an en-bloc resection, an ipsilateral thyroidectomy, and resection of involved surrounding tissues (10). However, because of lacking valid diagnostic methods to preoperatively distinguish malignancy from benign PT, it is still hard to perform proper procedures in the first operation, which may be responsible for most tumor recurrence. Although the overall patient prognosis is favorable, recurrences are frequent in PT, worsening the prognosis (3). Future progress of preoperative differential diagnosis is expected to guide the choice of the surgical procedures, thus contributing to a better prognosis of patients with PT.

Among most PT patients, the cause of death is often not tumor burden, but uncontrollable hypercalcemia caused by hyperparathyroidism. Many therapies are being developed to manage hypercalcemia in PT, especially in inoperable PT, including bisphosphonates, RANK ligand antibody, calcitonin, and calcimimetics (11). As the newest generation of calcimimetic, cinacalcet was proven to be effective in patients with inoperable parathyroid cancer by increasing the affinity of calcium-sensing receptors and reducing the secretion of parathyroid hormone (12).

## Conclusion

The annual scientific publications on PT scarcely changed during the last two decades. ^99m^Tc-MIBI imaging, parathyroidectomy, and gene expression are the most concerned topics in PT research. More efforts should be paid in gene expression pattern detection through preclinical research to clinical diagnosis and treatments.

## Data Availability Statement

The original contributions presented in the study are included in the article/supplementary material. Further inquiries can be directed to the corresponding author.

## Author Contributions

Conceptualization: XL. Data curation: ZZ. Formal analysis: ZZ; Methodology: ZZ and FX. Project administration: XL. Supervision: FX and XL. Validation: FX. Visualization: FX. Writing—original draft: ZZ. Writing—review and editing: FX and XL. All authors contributed to the article and approved the submitted version.

## Funding

This work was supported by the National Natural Science Foundation of China (grant no. 82073262) and the Hunan Province Natural Science Foundation (grant number 2019JJ40475).

## Conflict of Interest

The authors declare that the research was conducted in the absence of any commercial or financial relationships that could be construed as a potential conflict of interest.

## Publisher’s Note

All claims expressed in this article are solely those of the authors and do not necessarily represent those of their affiliated organizations, or those of the publisher, the editors and the reviewers. Any product that may be evaluated in this article, or claim that may be made by its manufacturer, is not guaranteed or endorsed by the publisher.
